# Editorial: Host-pathogen interaction in cestodes infection

**DOI:** 10.3389/fcimb.2023.1283267

**Published:** 2023-09-11

**Authors:** Jayaraman Tharmalingam, Klaus Brehm, Suman Kundu, Daniel Młocicki, Rodolfo Paredes

**Affiliations:** ^1^ Wisconsin Institute for Discovery, University of Wisconsin-Madison, Madison, WI, United States; ^2^ Institute of Hygiene and Microbiology, Julius-Maximilians-University of Würzburg, Würzburg, Germany; ^3^ Department of Microbiology, Immunology and Biochemistry, University of Tennessee Health Science Center (UTHSC), Memphis, TN, United States; ^4^ Department of General Biology and Parasitology, Medical University of Warsaw, Warsaw, Poland; ^5^ Laboratorio de Medicina Veterinaria, Escuela de Medicina Veterinaria, Facultad de Ciencias de la Vida, Universidad Andres Bello, Santiago, Chile

**Keywords:** cestode, echinococcus, cysticercosis, host - pathogen interactions, parasite antigen

Host-pathogen interaction is a complex process specifically during infection with multicellular parasites such as cestodes. Every year, millions of people become infected, and thousands of deaths occur due to this. The World Health Organization listed many of the cestode infections under Neglected Tropical Diseases (NTDs) to address the urgent need to prevent the infection. Number of the NTDs causes millions of Disability Adjusted Life Years (DALYs) and cost billions of dollars in morbidity and mortality associated with diseases every year (Engels and Zhou, 2020). For thousands of years, tapeworms co-evolved with many of its intermediate and definite hosts (including humans) and this complicated and undermined the host response to cestode infections. The complexity of cestodiases is one of the major reasons for a sustained infection burden worldwide, even though effective treatments are available. The most serious pathological changes during cestode infections are related formation space-occupying metacestodes in diverse of the hosts vital organs and such fatal infections cause neurocysticercosis, cysticercosis, coenurosis, echinococcosis, and sparganosis. In many cases metacestodes are formed by Vesicle Fluid (VF) filled in a space occupying Vesicle Tissue (VT).

Neurocysticercosis is a cestode *Taenia solium* metacestode infection of the human central nervous system. Cyst antigens differentially activate the host immune responses in endemic regions, and thousands of people are exposed to infective antigens, but very few people develop diseases (Jayaraman et al., 2011). Similarly, host immune responses that alter the nature of *Echinococcus* cyst antigens dominate the development of clinically pathogenic diseases. Understanding cestode cyst antigens and host immune responses against those antigens is vital for preventing and eliminating fatal cestode infections. The scientific research section “Host-pathogen interactions in cestode infections” in Frontiers in Cellular and Infections Microbiology and four of the published research articles in this section cover various aspects of metacestode antigens and host interactions in echinococcosis. Prevention of many of the NTDs is achievable by developing accurate and affordable diagnostic tests. In the case of cestodiases, including echinococcal diseases, diagnostics based on host humoral immune responses against metacestode antigens are highly effective for early diagnosis and treatment as well as for treatment effectiveness evaluation.

When the intermediate host swallow the infective eggs containing hexacanth larvae, these penetrate intestinal tissues, migrate, and undergo transformation into metacestode stage as a form of cyst (*Echinococcus, Taenia*), which may be found in different tissues and organs of the host. Host immune responses are an important determining factor for the establishment of cyst infections. Host recognition of cyst antigens and antigenic proteins vary based on many factors: cestode species, the location of cyst antigens, the excretory and secretory nature of the antigens, and host immune response modulated by and for cyst responses.

The nature of cyst antigens varies between different species of the cestode, and identification of species-prevalent and species-specific antigens improve the prevention of spread of the infection. Basharat et al., research on genetic variation of echinococcosis in an endemic region in Pakistan established the importance of identification of species and immunogenicity against infection. Most of the cyst infections were noticed in the liver and more than 85% of these infections are caused by *Echinococcus granulosus* sensu stricto. Authors reinstated other studies finding that the major source of cystic echinococcus around the world was domestic animal trades between many of the infection endemic countries.

Host immune response against cestodes did not depend on specific species, but within species metacestodes proteins played a major role in affecting host immune responses. Müller et al., in this section try to characterize the location-specific protein in metacestodes of *Echinococcus multilocularis*, a causative agent of alveolar echinococcosis. Authors found many parasite proteins differentially present between vesicle fluid and vesicle tissue, and they found more than 93% of proteins made up of AgB subunits. Characterization of antigen AgB has higher values, as AgB of *Echinococcus* is important for host immune modulation and plays a major role in serodiagnosis of *Echinococcus* infection. Also, authors showed that AgB proteins are involved in lipid transportation from the host to metacestodes to reduce the metabolic burden of the metacestode.

Cestode infections are preventable if they are diagnosed early in a community and when infection monitoring is carried out. Screening of seroprevalence of antigens in primary and intermediate hosts enhances the prevention of community spread of the infection. Kronenberg et al., developed a monoclonal antibody assay for diagnosis of different stage-specific antigens in *Echinococcus*. Clinical relevance of Em18 antibodies is important and is used to interpret the outcome of the treatment for alveolar echinococcus (Hotz et al., 2022). Kronenberg et al., showed that their monoclonal antibodies (mAb Eg2) are reactive to antigen from both the germinal layer and the protoscoleces in both alveolar and cystic echinococcosis.

Host cellular immune response against parasites is an important factor to limit the infection and eliminate the parasite. In the case of alveolar echinococcosis, however, the immune response of the intermediate host is gradually skewed towards an immunosuppressive environment during infiltrative parasite growth within the liver. Kaethner et al. report how this immune response might influence the differentiation of the *Echinococcus multilocularis* metacestode towards the next larval stage, the protoscolex. These authors showed that *E. multilocularis* expresses a complex TGF-β signaling system which responds to both host and parasite TGF-β ligands. Furthermore, they demonstrate that functional parasite TGF-β signaling is necessary for protoscolex formation initiated by stem cells located in the germinative layer. Since high concentrations of host TGF-β are accumulating around parasite lesions at late stages of alveolar echinococcosis, the immune response of the intermediate host might therefore be a trigger telling the parasite when to differentiate into the infective form for the definitive host.

Within this Research Topic, all four articles established the importance of host-parasite interactions in echinococcosis, which continues to be an important health and life-threatening problem for many people, especially in endemic areas. Researchers indicate the usefulness of serological diagnostic and molecular methods in the identification, diagnosis and prevention of infections. They point to the molecular mechanisms underlying the formation of larvae, and those that are key to better understanding the biology of cestodes and their interaction with the host.

## Author contributions

JT: Writing – original draft, Writing – review & editing. KB: Writing – original draft, Writing – review & editing. SK: Writing – original draft, Writing – review & editing. DM: Writing – original draft, Writing – review & editing. RP: Writing – original draft, Writing – review & editing.
